# Corrosion Performance of Fe-Cr-Ni Alloys in Artificial Saliva and Mouthwash Solution

**DOI:** 10.1155/2015/930802

**Published:** 2015-05-06

**Authors:** J. Porcayo-Calderon, M. Casales-Diaz, V. M. Salinas-Bravo, L. Martinez-Gomez

**Affiliations:** ^1^CIICAp, Universidad Autónoma del Estado de Morelos, Avenida Universidad 1001, 62209 Cuernavaca, MOR, Mexico; ^2^Instituto de Ciencias Físicas, Universidad Nacional Autónoma de México, Avenida Universidad s/n, 62210 Cuernavaca, MOR, Mexico; ^3^Instituto de Investigaciones Eléctricas, Avenida Reforma 113, Colonia Palmira, 62490 Cuernavaca, MOR, Mexico; ^4^Corrosion y Protección (CyP), Buffon 46, 11590 México City, DF, Mexico

## Abstract

Several austenitic stainless steels suitable for high temperature applications because of their high corrosion resistance and excellent mechanical properties were investigated as biomaterials for dental use. The steels were evaluated by electrochemical techniques such as potentiodynamic polarization curves, cyclic polarization curves, measurements of open circuit potential, and linear polarization resistance. The performance of steels was evaluated in two types of environments: artificial saliva and mouthwash solution at 37°C for 48 hours. In order to compare the behavior of steels, titanium a material commonly used in dental applications was also tested in the same conditions. Results show that tested steels have characteristics that may make them attractive as biomaterials for dental applications. Contents of Cr, Ni, and other minor alloying elements (Mo, Ti, and Nb) determine the performance of stainless steels. In artificial saliva steels show a corrosion rate of the same order of magnitude as titanium and in mouthwash have greater corrosion resistance than titanium.

## 1. Introduction

Biomaterials are materials used in the manufacture of devices that interact with biological systems and coexist for a long period of service with minimal failure. They are widely used for repairing or replacement of components of the tissue-skeletal system such as bones, joints, and teeth. Suitable materials for implants are those endured by the body and they should be able to withstand cyclic loading in the aggressive environment of the body. Metals and alloys have been widely used as biomaterials providing the mechanical strength and corrosion resistance required. Biomaterials are generally made of one of these three types of materials: austenitic stainless steel, chromium-cobalt alloys, and titanium and its alloys.

Oral environment is suitable for the formation of corrosion products. The mouth is wet and it is continually exposed to temperature fluctuations. Foods and beverages cause important changes in environmental chemistry. The food and fluid ingested have wide ranges of pH. Acids are released during the decomposition of foodstuffs. Food remains often adhere tenaciously to metal restorations providing a localized condition favoring an accelerated reaction between oral media and metal or alloy [1]. One of the fundamental conditions of any metal used in the mouth is that they should not produce corrosion products that are harmful to the person. Corrosion is a chemical or electrochemical process by which metal is attacked by ordinary substances, resulting in partial or complete dissolution, deterioration, and weakness. Corrosion causes catastrophic disintegration of metals. Some metallic elements are completely safe but some can form dangerous ions or even toxic compounds. Additionally, degradation of an alloy should be limited in order to guarantee its life [2]. Various sulfides such as hydrogen sulfide or ammonium compounds corrode silver, mercury, and similar metals found in an amalgam. Water, oxygen, and chloride ions are in saliva and contribute to corrosion attack. The presence of various acids such as phosphoric, acetic, and lactic acid can lead to corrosion having the appropriate concentration and pH in the environment. Specific ions can play an important role in the corrosion of certain alloys; for example, oxygen and chlorine ions are involved in the corrosion of amalgams in the tooth interface and within the alloy. Therefore, it is important to consider the use of materials with high corrosion resistance in order to withstand the environments of the oral cavity.

Titanium and titanium alloys are widely used for many biomedical applications due to their low density, excellent biocompatibility, corrosion resistance, and mechanical properties. In the passive state, these alloys are not completely stable and under certain circumstances the passive film breaks down producing localized corrosion. This fault calls to modify the material surface to increase the corrosion and wear resistance without affecting their mechanical properties [3]. Austenitic stainless steel, specially type AISI 316-L, is the most widely used steel for implants, including orthodontic treatments because of its low cost, ease to fabricate, and welding if compared to Co-Cr and Ti alloys. Corrosion resistance is the most important characteristic of austenitic stainless steel due to formation of a passive film of Cr_2_O_3_ on its surface [4, 5]. 316-L type stainless steel has an acceptable corrosion resistance, biocompatibility, strength, and fatigue resistance that makes it a desirable material as biomaterial [6].

In recent years, there has been a rapid increase in the utilization of fluorinated prophylactic gels and mouthwash in the odontology field. The negative influence of fluoride on the corrosion of titanium and its alloys has been reported earlier [7–9]. Similarly, fluoride ions are aggressive ions that degrade the protective oxide layer formed on a stainless steels surface [10]. On the other hand, many mouthwashes contain both fluorine ions and hydrogen peroxide, and therefore the corrosion performance of biomaterials can be affected by the combined presence of both compounds. Antiseptic rinses are commonly used with antiplaque, anticaries, antibacterial, and whitening properties. Biomaterials and various surrounding tissues may get exposed to antiseptic rinses for several minutes every day, depending upon usage habits.

The objective of the present study was to evaluate the corrosion behavior of different austenitic stainless steels widely utilized in corrosive environments at high temperatures for dental applications in artificial saliva and mouthwash solution containing NaF and H_2_O_2_. The study was conducted using conventional electrochemical techniques.

## 2. Experimental Procedure

### 2.1. Materials

Stainless steels are divided into five families, each with its own microstructure, alloying elements, and specific mechanical properties. These stainless steels are classified as ferritic, austenitic, duplex, martensitic, and precipitation hardened. In particular, austenitic steels are the largest family of stainless steels; they cannot be hardened by heat treatment, are nonmagnetic, and have a face-centered cubic structure; in addition they have excellent ductility and good weldability. Six different austenitic stainless steels widely utilized in corrosive environments at high temperatures were evaluated.  Table 1 shows the materials evaluated and the limits of chemical composition as established by ASTM A213/A213M [11]. From Table 1 it can be seen that the contents of C, Mn, P, S, and Si are the same, and then it is possible that the stainless steels performance is based on the content of either Cr and Ni or other minor elements (Mo, Nb, and Ti). For comparison purposes commercial pure titanium (CP-Ti Grade 1) was also evaluated.

### 2.2. Sample Preparation

Test specimens were cut in squares of 5.0 × 5.0 × 3.0 mm using a diamond tipped blade. For electrical connection, specimens were spot-welded to a Ni20Cr wire and then mounted in thermosetting resin. Sample surfaces were metallographically polished; the grinding process began with 120-grit sandpaper down to 1200-grit sandpaper. After polishing, the materials were washed with distilled water, and after with ethanol in an ultrasonic bath for 10 minutes. In this condition the materials were used as working electrode (WE).

### 2.3. Corrosive Electrolytes

The most important fluid in the oral environment is the natural saliva. However, the unstable nature of natural saliva makes it unsuitable for* in vitro* studies and instead artificial saliva is used. In this work the corrosion performance of materials was evaluated in artificial saliva proposed by Duffó and Quezada-Castillo [12].  Table 2 reports the chemical composition of artificial saliva. Similarly, materials were evaluated in mouthwash widely used for hygiene purposes. Chemical composition of mouthwash reported by the manufacturer indicates it contains water, alcohol 8.8%, sorbitol, hydrogen peroxide 2%, sodium fluoride 0.022% (100 ppm de fluorine), PEG hydrogenated castor oil, sodium saccharin 0.119%, sucralose, phosphoric acid, disodium phosphate, poloxamer 407, flavor, and menthol. Test conditions were pH 6.5 and 37°C temperature, which is equivalent to human body temperature.

### 2.4. Electrochemical Tests

Electrochemical tests were carried out using an ACM instruments zero-resistance ammeter (ZRA) coupled to a personal computer. A typical three-electrode arrangement was used where the reference electrode (RE) was a saturated calomel electrode (SCE, 0.242 V versus SHE) and the counter electrode (CE) was a high-purity graphite rod (Pt cannot be used because it decomposes H_2_O_2_). All potentials described in the text are relative to the SCE, unless stated differently. The volume of solution in the cell was 100 mL, following the recommendation V/A specified in ASTM G31-90 [13]. To determine the corrosion resistance of materials, corrosion current density (*I*_corr_) was calculated by potentiodynamic polarization test from −400 mV to 1500 mV with respect to open circuit potential (*E*_corr_) according to standard ASTM G5-94 (reapproved 2004) [14] and ASTM G-3-89 (reapproved 2004) [15]. These tests allow determining the corrosion potential and the corrosion rate by extrapolation of the Tafel slopes from the curves obtained. For all materials, potentiodynamic polarization tests were performed at a sweep rate of 1 mV/s. Before starting the test, samples were left to stabilize for 20 minutes. The current density values (*I*_corr_) and anodic and cathodic slopes and corrosion potential (*E*_corr_) were calculated using the extrapolation Tafel method considering an extrapolation potential of ±250 mV around the value of the corrosion potential (*E*_corr_).

A cyclic polarization technique was used to evaluate pitting corrosion resistance of all materials tested according to the ASTM F2129-01 [16] and ASTM G3-89 [15]. The working electrode was stabilized at the free corrosion potential for an hour, and then a potential sweep (0.166 mV/s) was made in the anodic direction until the potential reaches a predetermined value. At this point, the scan direction is reversed until the hysteresis loop closes. To assess the ability of the different materials to form a protective oxide scale on their surfaces upon immersion in the simulated body fluid environment, free corrosion potential as a function of time of the working electrodes *E*_corr_ was measured versus a SCE for 50 hours. Linear polarization curves were obtained by polarizing the specimens from −30 to 30 mV with respect to the free corrosion potential value, *E*_corr_, at a scanning rate of 10 mV/min; measurements were made for 50 hours. After testing, corroded specimens were analyzed in a DSM 960 Carl Zeiss scanning electronic microscope (SEM).

## 3. Results and Discussion

### 3.1. Potentiodynamic Polarization Curves

**Figure 1:**
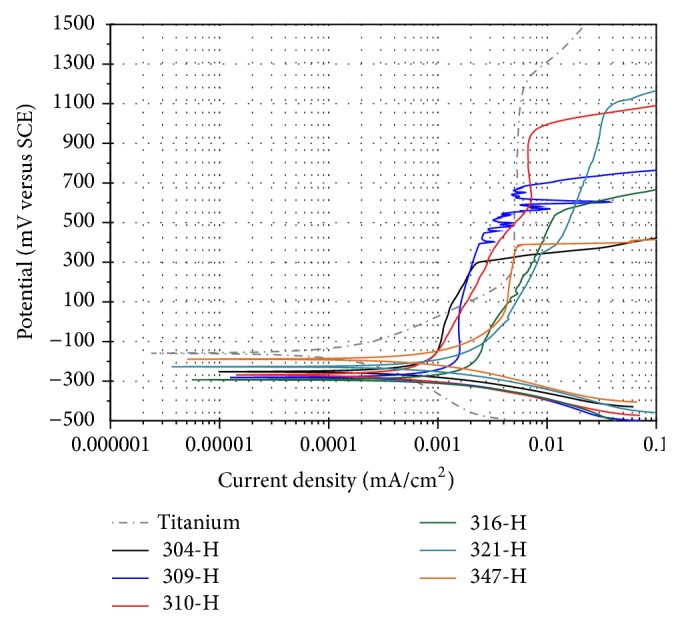
Polarization curves of materials in artificial saliva at 37°C,* dE/dt* = 1.0 mV/s.

Figure 1 shows potentiodynamic polarization curves of materials evaluated in artificial saliva at 37°C. It can be observed that titanium has the nobler *E*_corr_ and stainless steels the most active *E*_corr_, but, at potentials above *E*_corr_, stainless steels reach a pseudopassivation zone at lower potentials. Above its corrosion potential, titanium shows a passive region from 250 mV to 1200 mV; this is due to the growth of a protective TiO_2_ layer on its surface. Stainless steels show virtually the same comportment. Small variations observed in stainless steels may correspond to the variations in their chemical composition. Generally it can be seen that above its corrosion potential all steels show a tendency to slow down corrosion by increasing the applied potential. This trend seems to correspond to an attempt to develop a passive region because of the formation of a Cr_2_O_3_ protective film on its surface. Extending this apparent passive region behavior is different for each one of steels. At end of the passive region an abrupt increase is observed in the current density that may correspond to the onset of pitting on the surface of steels. The active region of polarization curves is due to the electrochemical reaction of the sample in the electrolyte medium and formation of a porous or defective oxide layer, whereas the passive region is associated with the formation of one or more protective oxide films.

Particularly, steel 309-H clearly shows the breakdown and repassivation of the protective layer before reaching its pitting potential. Other steels do not show this tendency. This could be because a potential sweep of 1 mV/s was applied; possibly slower sweep rates (0.167 mV/s) could define the passive region and more precise values of pitting potential and other parameters of interest to biomaterials. It is to be noted that steels 316-H, 321-H, and 347-H, with addition of Mo, Ti, and Nd, are materials whose apparent passivation areas are located at higher current densities compared to those containing only Cr and Ni (304-H, 309-H, and 310-H). In general, stainless steels showed the highest corrosion rates, and the material with greater corrosion resistance was titanium.  Table 3 shows electrochemical parameters obtained from polarization curves shown in Figure 1.

**Figure 2:**
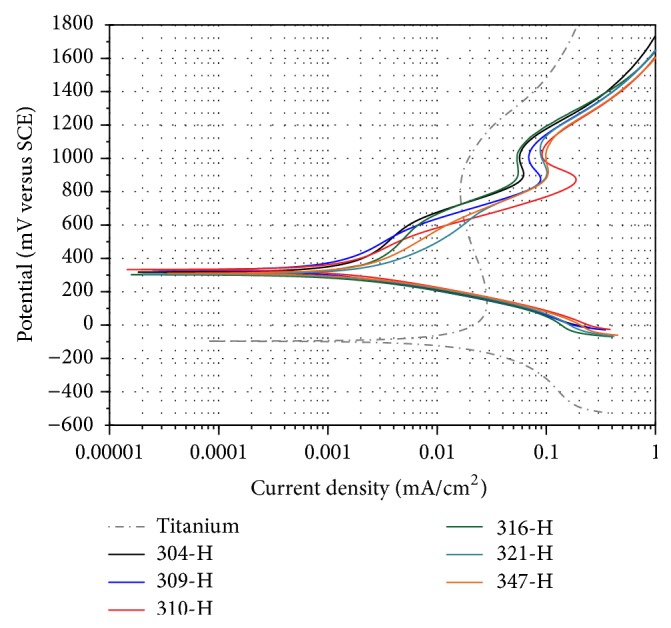
Polarization curves of materials in mouthwash at 37°C,* dE/dt* = 1.0 mV/s.

Figure 2 shows potentiodynamic polarization curves of materials evaluated in mouthwash at 37°C. Titanium shows the most active *E*_corr_ but quickly develops a passive zone at more anodic potentials. Stainless steels having similar *E*_corr_ values show a more homogeneous behavior than that observed in the presence of artificial saliva (Figure 1). Moreover, their anodic branches are almost the same with a passive region between 850 and 1050 mV. Small variations observed correspond to differences in chemical composition of each material. Stainless steels show lower corrosion rates and titanium the highest.  Table 4 shows the electrochemical parameters obtained from the polarization curves of Figure 2.

**Figure 3:**
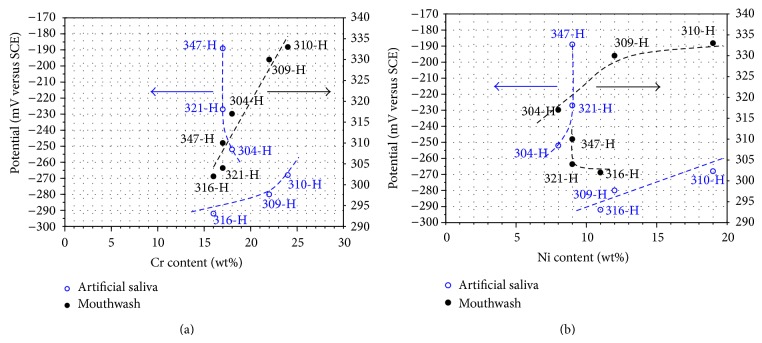
Effect of Cr and Ni content of stainless steels on *E*_corr_ values.

**Figure 4:**
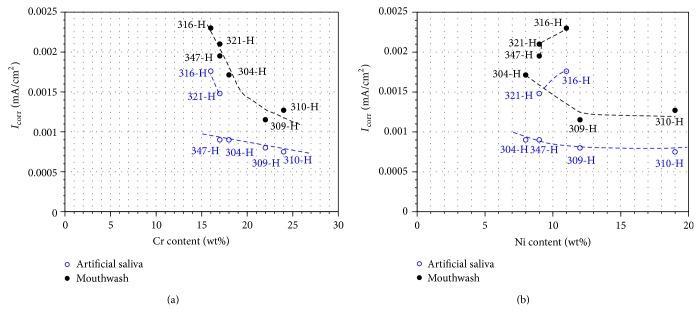
Effect of the Cr and Ni content of stainless steels on *I*_corr_ values.

Although stainless steels have the same tendency, its particular position on the graph and the particular values of their electrochemical parameters depend on its chemical composition. Thus in Figure 3 the effect of Cr and Ni content on *E*_corr_ values is shown and in Figure 4 the effect of Cr and Ni content on *I*_corr_ values is also shown. The Cr and Ni values plotted in both figures correspond to the minimum content of these elements in stainless steels according to ASTM A213/A213M-04 (Table 1).

In artificial saliva (Figure 3(a)) steels 316-H, 309-H, and 310-H show that Cr contents above 16%, *E*_corr_ of steels, become nobler. However, steels 304-H, 321-H, and 347-H are out of this trend, showing a nobler *E*_corr_ having a Cr content between 17 and 18%. This discrepancy is associated with the Ni content of these steels (Figure 3(b)). It is observed that those steels with less Ni content exhibit a nobler *E*_corr_. Nevertheless, the difference in Ni content between steels 304-H, 321-H, and 347-H does not justify such a significant difference among their *E*_corr_ values. This can be explained because steels 321-H and 347-H contain Ti and Nb, respectively, which are added as stabilizing elements. Nb seems to have a favorable effect on *E*_corr_ of steel 347-H, followed by Ti in steel 321-H. This is consistent with the behavior of Ti according to Figure 1 where it was noted that Ti has a nobler *E*_corr_ than that of stainless steels.

Figure 4(a) shows a direct relationship between Cr content of stainless steels and corrosion rate. Steels having higher Cr content have small values of *I*_corr_. It is also seen that, in the particular case of 316-H and 321-H steels, they have higher *I*_corr_ values than steels having similar content of Cr and Ni like 347-H and 304-H steels. In these cases, these steels contain additional elements such as Mo for the case of 316-H and Ti in case of 321-H as shown in Table 1. This indicates that the incorporation of these elements brings a detriment in the corrosion resistance of these steels in artificial saliva environments. Furthermore, it is noted that even though 347-H contains Nb, it does not significantly affect the steel corrosion resistance. Regarding the Ni content a similar trend is observed as that described for the Cr content. Therefore it can be established that the corrosion potential (*E*_corr_) of stainless steels is affected by the Cr and Ni contents. Cr has a beneficial influence on the passive behavior of steels with lower Ni content. The addition of stabilizing elements such as Nb and Ti has a greater effect than the increase in the concentration of Cr. On the other hand, the corrosion rate of steels (*I*_corr_) is directly related to the content of Cr and Ni in the alloy. Higher content of both elements increases the corrosion resistance; however, addition of Mo and Ti negatively affects the corrosion resistance.

In mouthwash (Figure 3(a)) a clear effect of Cr content of stainless steels on *E*_corr_ values is observed. Higher content of Cr in steels results in nobler corrosion potentials. However, observing the Ni content (Figure 3(b)), only in steels 304-H, 309-H, and 310-H, the tendency of greater *E*_corr_ with higher Ni content is maintained. In case of steels 347-H, 321-H, and 316-H, this trend is not followed. Examining the chemical composition for the late steels it can be seen that they contain Nb, Ti, and Mo, respectively. Then the addition of Mo creates a greater impact in decreasing *E*_corr_ of 316-H steel (high Cr and Ni content compared to 321-H and 347-H), followed by Ti in 321-H (same content of Cr and Ni as in 347-H). This is consistent with the results of the polarization curves where it was observed that Ti showed the more active behavior (Figure 2).

Regarding their corrosion rate, Figure 4(a) shows a direct relationship between the Cr content of the stainless steels and corrosion rate. Steels with higher content of Cr have lower *I*_corr_ values. Concerning the effect of the Ni, Figure 4(b) shows that in case of steels 304-H, 309-H, and 310-H there is a link (not so marked as in the case of Cr) between higher Ni content and lower *I*_corr_. In case of steels 316-H, 321-H, and 347-H the trend is not followed. This can be due to the content of Mo, Ti, and Nb affecting adversely the corrosion resistance. Therefore an increase in the Cr and Ni content favors a nobler behavior (>*E*_corr_), the effect being greater with increasing Cr; addition of Mo, Nb, and Ti causes more active behavior. The worst performance observed by titanium is because the presence of fluorine ions and H_2_O_2_ has a negative influence on the corrosion rate. It is known that the presence of fluoride ions causes surface films to become less stable and eventually a breakdown of the film may occur [17]. On the other hand, the presence of H_2_O_2_ causes the dissolution of titanium, formation of a titanium oxide on the metal surface, and Ti catalyses decomposition of peroxide [18].

### 3.2. Cyclic Polarization Curves

**Figure 5:**
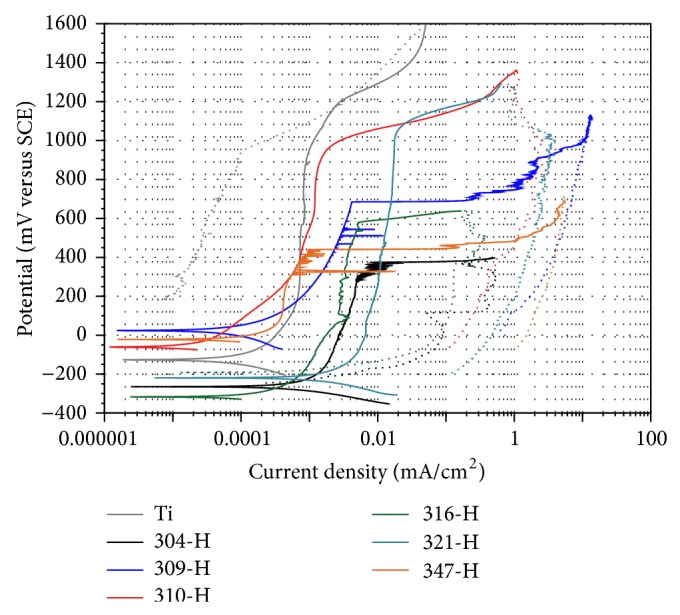
Cyclic polarization curves recorded for materials in artificial saliva at 37°C,* dE/dt* = 0.167 mV/s.

**Figure 6:**
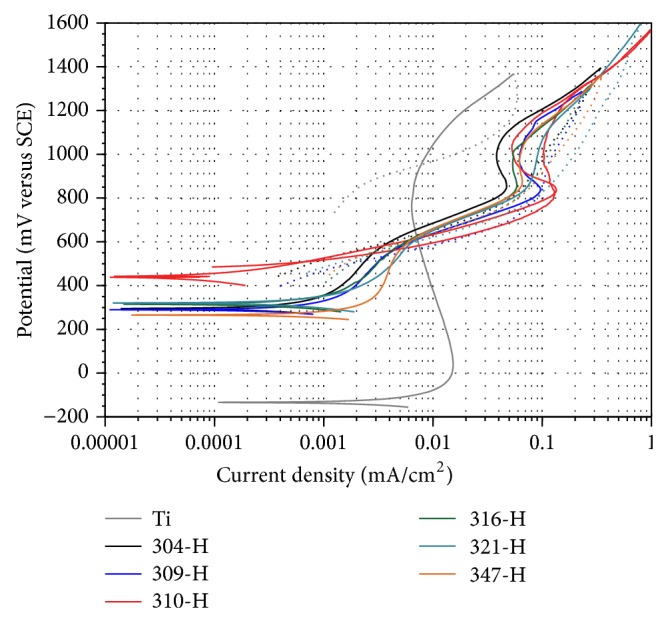
Cyclic polarization curves recorded for materials in mouthwash solution at 37°C,* dE/dt* = 0.167 mV/s.

Because any material or alloy proposed for use as a biomaterial must exhibit excellent resistance to localized corrosion, cyclic polarization measurements were performed in order to determine the tendency of the alloys to undergo pitting or crevice corrosion when submerged in the electrolyte solutions. A plot of potential versus log of the current density reveals the tendency of the material to undergo localized attack. Differences between the forward and reverse scans result in hysteresis loops. If the current density during the reverse scan is higher than that for the forward scan at any given potential (positive hysteresis loop), the area under the loop indicates the amount of localized corrosion incurred by the material. Conversely, if the current density during the reverse scan is less than that for the forward scan at any given potential (negative hysteresis loop) it indicates high resistance to localized corrosion. Two important potentials are also used to characterize the hysteresis loop, the pitting nucleation, or breakdown potential (*E*_np_) and the protection potential (*E*_pp_).  *E*_np_ is defined as the potential at which the pitting or crevice corrosion or both will initiate and propagate and in the polarization curve appears as an abrupt increase in the anodic current density at the point where the passive zone ends. Pitting is characterized by a rapid increase in current with a very small change in potential. Above this potential, pits initiate and propagate. An increase in the resistance to pitting corrosion is associated with a shift of *E*_np_ to nobler values. *E*_pp_ is defined as the potential at which the forward and the reverse scans intersect. At this potential, localized attack stops and the current decreases significantly. This value is always lower than the *E*_np_ value [19]. Figures 5 and 6 show the cyclic polarization curves for materials tested in artificial saliva and mouthwash solution, respectively. In general by increasing anodic polarization, the formation and repair of an anodic film are observed. Nevertheless, the irreversible breakdown of the passive film is clearly identified by the abrupt increase of the current density. The differences in composition of the alloys affected the polarization and the passivation behaviors of tested materials.

Figure 5 shows that in artificial saliva the anodic branch of the cyclic polarization curves of all materials exhibits an active-passive transition. It is known that by increasing anodic polarization several processes may take place on the materials' surface, namely, the formation and repair of an anodic film, water oxidation leading to electrogenerated oxygen bubbles, gas evolution from the oxidation reaction of some entities (e.g., Cl_2_ production through 2Cl^−^ → Cl_2_ + 2e^−^), and the onset of the breakdown at critical potential [20]. Ti, 310-H, and 321-H steels showed the largest passive zone compared to that shown by the rest of the stainless steels. 304-H, 309-H, 316-H, and 347-H steels show a passive zone with minor disturbances. As reported by other studies, these disturbances are created because of the breakdown and regeneration of the passive layer formed [21–23]. In the passive-pitting zone (*E*_np_), Ti, 310-H, and 321-H steels showed a slow increase in current with a very small change in potential. However, the rest of the stainless steels showed an abrupt increase; this indicates both a greater growth and propagation of pitting. Ti has higher resistance to localized corrosion because the current density during the reverse scan is lower than that for the forward scan. However, stainless steels showed a positive hysteresis loop and the reverse scan continues at potentials below the corrosion potential. Only 304-H and 316-H steels showed an intersection with the forward scan but at a potential near to its corrosion potential. These features indicate a high susceptibility to pitting of stainless steels in artificial saliva.

Figure 6 shows that Ti has more active behavior and 321-H the noblest in mouthwash solution. Ti showed the largest passive zone, with *E*_pp_ close to its *E*_np_ (around 930 mV). On the other hand, stainless steels showed a similar behavior; a small passive zone was observed at potentials away from its corrosion potential. The *E*_np_ values showed slight variations with values around 1000–1070 mV. Similar behavior had the *E*_pp_ values around 850–900 mV. In all cases, a slow increase in current with a small change in potential was observed after *E*_np_ zone. This indicates that, in mouthwash solution, both the growth and propagation of pitting are less than those observed in artificial saliva.

**Figure 7:**
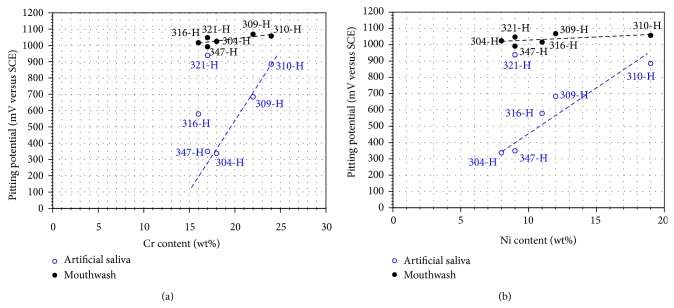
Effect of the Cr and Ni content of stainless steels on pitting potential.

In order to determine the effect of chemical composition of stainless steels with respect to the pitting potential, Figure 7 shows the relation of the chromium and nickel content on the *E*_np_ value. The lowest Cr and Ni content as reported by ASTM A213/A213M were plotted. It is evident that the effect is more noticeable in artificial saliva than that observed in mouthwash solution. In artificial saliva it is observed that the pitting potential increases by increasing the chromium content. However, 347-H, 316-H, and 321-H have a lower chromium content and do not show the same behavior. This is because Nb, Mo, and Ti content affect the pitting potential. The effect is more marked with the addition of Ti; that is, the addition of Ti to steel 321-H gives it greater resistance to pitting corrosion than that provided by the addition of Mo to 316-H. On the other hand, it is observed that the nickel content also affects the pitting potential. In this case, the Nb and Mo content have not a significant effect; however, the titanium content shows a marked effect. In mouthwash solution, it is observed that by increasing chromium and nickel content the pitting potential shows a slight increase, and in this case, Nb, Mo, and Ti content have not a significant influence.

### 3.3. Free Corrosion Potential Curves

**Figure 8:**
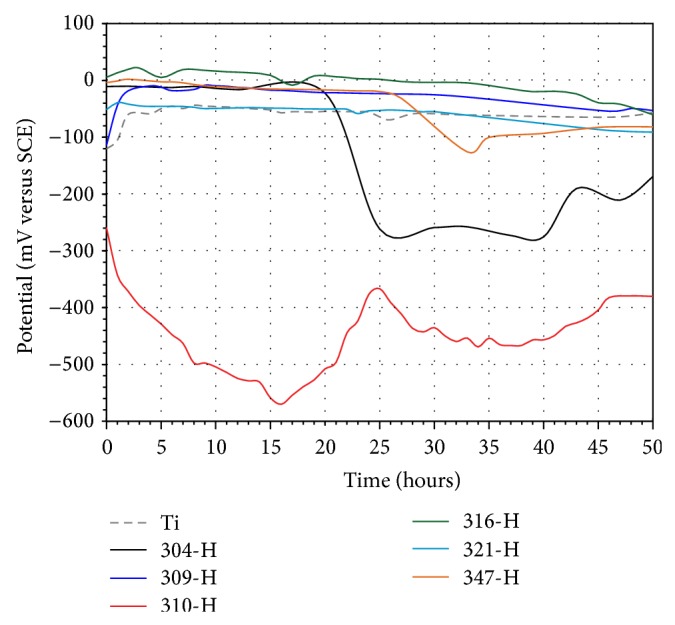
*E*_corr_ values after testing time for materials in artificial saliva at 37°C.

**Figure 9:**
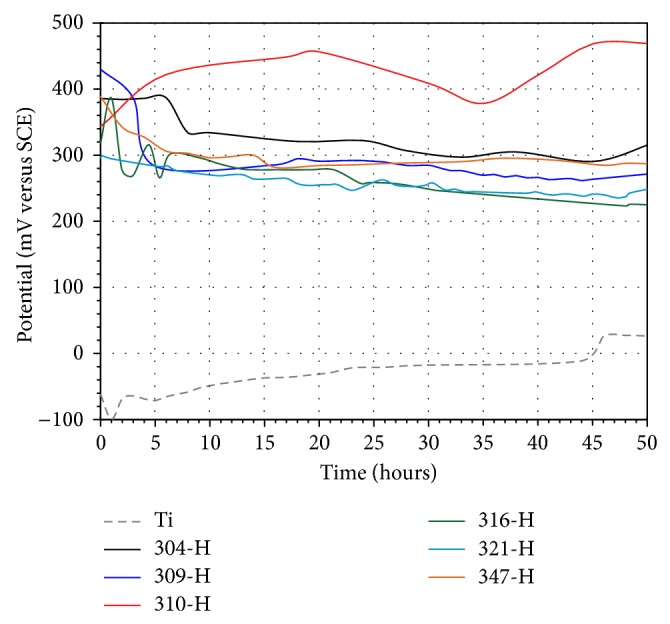
*E*_corr_ values after testing time for materials in mouthwash solution at 37°C.

Establishing the chemical interaction of a biomaterial with the body fluid environment is essential in order to understand its stability in the human body. *E*_corr_ gives a relative thermodynamic rating of a metal or alloy in a given environment. A rise of *E*_corr_ in the positive direction indicates the formation of a passive film, and a steady *E*_corr_ indicates that the film remains intact and protective, but a drop of *E*_corr_ in the negative direction indicates breaks in the film, dissolution of the film, or no film formation [24]. Figures 8 and 9 show the variation of *E*_corr_ values as a function of time for materials tested in artificial saliva and mouthwash solution, respectively.

According to the concepts stated above, it can be seen that Ti shows a passive behavior in artificial saliva. In the first 2 hours, Ti shows a significant increase in its *E*_corr_ values and then a slow increase until the end of the test. This passive tendency is due to the formation of a protective layer on its surface. Stainless steels showed an active behavior; in particular 309-H, 316-H, and 321-H showed a more stable behavior compared to 304-H, 310-H, and 347-H. 304-H and 347-H steels showed an abrupt decrease in its *E*_corr_ values at 20 and 27 hours of test, respectively, and then an increase in its *E*_corr_ values. 310-H steel showed continuous increments and decrements in *E*_corr_ values throughout the test and only after 45 hours showed a stable trend. The abrupt drop in *E*_corr_ values may be due to the susceptibility of these materials to pitting corrosion in chloride-rich environments, but it is evident that, in all cases, materials exhibit an immediate tendency to self-healing of its passive layer. The passivity of the alloys depend on the rates of several processes; such as formation of oxide at the metal/oxide interface, ionic transport across oxide, and dissolution of oxide at the oxide/electrolyte interface [25]. Steel 321-H showed *E*_corr_ values very similar to Ti. 316-H steel presented nobler *E*_corr_ values; this is expected because the presence of Mo in the steel provides greater resistance to corrosion in chloride-rich environments.

In mouthwash, Ti showed a more active behavior than that observed in artificial saliva. After the first hour Ti shows a slight decrease in its *E*_corr_ values and then a steady increase until the end of the test. Titanium is prone to become covered with an oxide film in noncomplex solutions of pH ranging from 3.5 to 7.5. However, the initial behavior could be explained because the fluoride ions caused a localized corrosion and partial dissolution of the protective film. The subsequent passive trend observed could be explained because of the combined effect of the fluoride ion with the organic active ingredients of the mouthwash solution as it has been suggested [25]. Stainless steels showed a passive-active behavior in mouthwash solution. An active trend was observed in the first 45 hours and subsequently a passive trend. 310-H showed the noblest behavior, with a continuous increment in its *E*_corr_ values until 20 hours of immersion and then a decrease until 35 hours, and finally a continuous increase. This material contains the highest content of chromium and nickel of the stainless steels evaluated. Regarding stainless steels, it is interesting to note that the trend observed in the *E*_corr_ values in mouthwash is completely opposite to the trend observed in artificial saliva (Figure 8). Steels that showed nobler behavior in artificial saliva in mouthwash showed the most active behavior and vice versa. The addition of Mo and Ti seems to be not beneficial for obtaining nobler *E*_corr_ values in stainless steels.

### 3.4. Linear Polarization Curves

**Figure 10:**
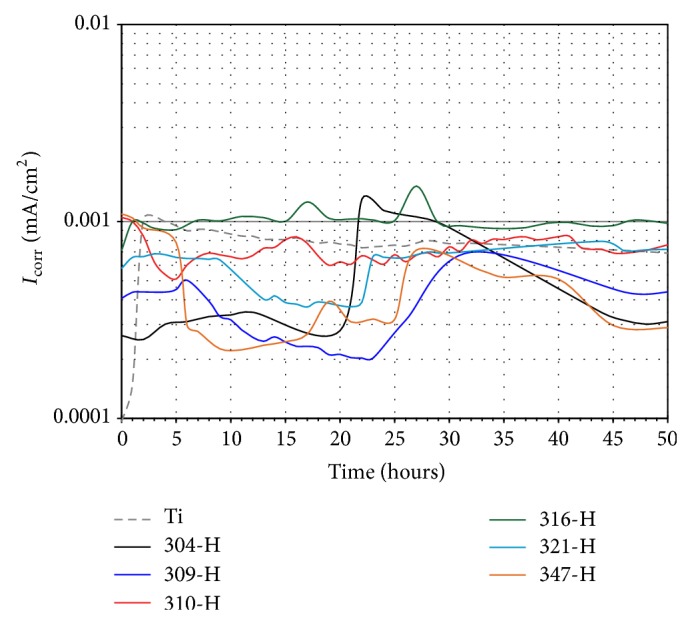
Change of *I*_corr_ values with time of materials in artificial saliva at 37°C.

**Figure 11:**
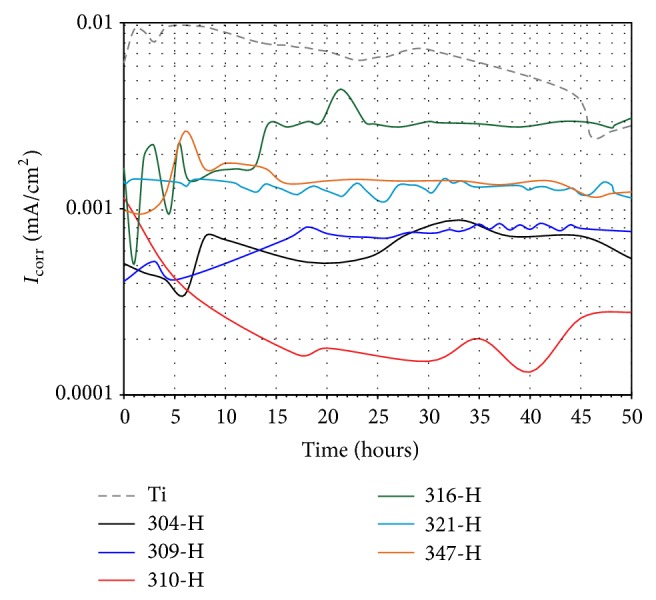
Change of *I*_corr_ values with time of materials in mouthwash solution at 37°C.

Once polarization resistance is determined, calculation of *I*_corr_ requires knowledge of the Tafel constants, and these constants are determined from experimental polarization curves. However, in the absence of the Tafel constants values, an approximation is sometimes used, and the expected error in the calculated value of *I*_corr_ should be less than a factor of two [26]. When the polarization resistance values are in the same order of magnitude, it is necessary to use more precise values of these constants in order to perform a reliable analysis of the results [27]. Progress of *I*_corr_ obtained by linear polarization measurements over time for materials evaluated in artificial saliva and mouthwash solution is showed in Figures 10 and 11. Data was obtained from the polarization resistance measurements using Stern-Geary expression:

(1) $$\begin{array}{c} i_{\text{corr}}=\frac{b_{a}b_{c}}{2.303R_{p}\left(b_{a}+b_{c}\right)}, \end{array}$$

where the *b*_*a*_ and *b*_*c*_ values were those reported in Tables 3 and 4.

It is observed that, in artificial saliva in the first 35 hours, materials showed constant fluctuations in their *I*_corr_ values, and then a steady behavior was displayed (Figure 10). This performance is consistent with *E*_corr_ measurements, where an active-passive condition was observed. Ti showed an abrupt increase in its *I*_corr_ values in the first 2 hours and then stable decrements until the end of the test. This can be explained because of dissolution of the passive layer and then self-healing of the titanium surface. The excellence of titanium as biomaterial is due to the passive layer that forms on its surface which protects it from electrochemical attack in the human body. This layer is composed of amorphous titanium oxides from Ti_2_O to TiO_2_, having variable thickness between 0.5 and 10 nm according to the treatment, surface finish, corrosive medium, and so forth [28–30]. It is reported that the passivation layer is formed naturally after a few milliseconds of contact with a medium with oxygen [31]. Stainless steels showed that, in long time immersion test, the lowest *I*_corr_ values were exhibited by 304-H, 309-H, and 347-H, and the higher *I*_corr_ values were showed by 316-H, 310-H and 321-H, having similar *I*_corr_ values to Ti at the end of the test.

Figure 11 shows *I*_corr_ values for materials in mouthwash solution. All materials showed a more stable behavior than that exhibited in artificial saliva. Ti showed an increase in its *I*_corr_ values in the first hour and then a steady decline until the end of the test. According to *I*_corr_ values, the corrosion resistance of the stainless steels is in the following order: 310-H > 304-H > 309-H > 321-H ≈ 347-H > 316-H. This means that steels with Ti, Nd, or Mo had the highest corrosion rates.

**Figure 12:**
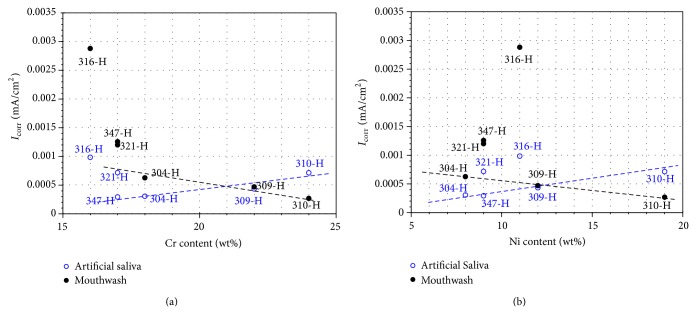
Effect of Cr and Ni content of stainless steels on *I*_corr_ values for long time tests.

Figure 12 shows the average *I*_corr_ values corresponding to the last 5 hours of testing for each material according to its minimum content of Cr and Ni as reported in ASTM A213/A213M.

In artificial saliva it is observed that the corrosion resistance of stainless steels increases with the contents of chromium and nickel. Steels 316-H and 321-H do not follow this trend; both exhibit higher corrosion rates than expected according to trends estimated by their Cr and Ni contents. Both steels contain additional elements such as Mo (316-H) and Ti (321-H). This suggests that the presence of these elements causes a detriment to corrosion resistance in environments of artificial saliva. On the other hand, 347-H steel also contains an additional element (Nb) and its presence did not affect the corrosion resistance. Similar results were obtained in potentiodynamic polarization tests. However in this case, the effect of the Cr and Ni content was opposite to that observed in the potentiodynamic polarization tests.

**Figure 13:**
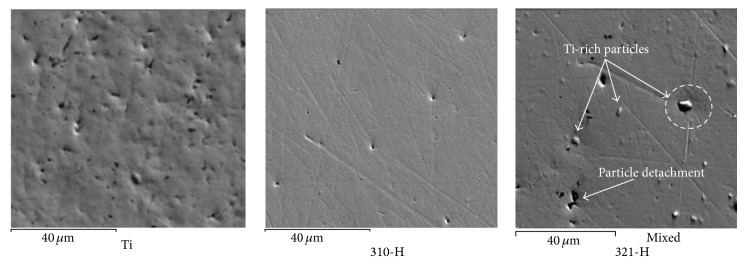
Superficial aspect for Ti, 310-H, and 321-H stainless steel after corrosion test in artificial saliva.

Although electrochemical methods are extremely useful in studying corrosion processes, they alone do not provide enough information to elucidate the mechanism of the system under study. Therefore the use of complementary techniques has been suggested, that is, scanning electron microscopy (SEM) and auger electron spectroscopy (AES), among others in order to clarify both the morphology of the attack and the chemical composition and distribution of the elements present. Combination of these methods provides the information for understanding the reactions occurring on the surface [32]. Consequently, in this study the surface of the working electrodes was studied using scanning electron microscopy (SEM) in order to clarify the morphology of attack.  Figure 13 shows the superficial aspect of titanium and 310-H and 321-H stainless steel after the corrosion test in artificial saliva.

Surface appearance shown by Ti after the corrosion test shows a smooth surface with the presence of slight pitting. Pit location is associated with lines resulting from surface preparation. This is consistent with studies indicating that any imperfections in the material surface can modify its resistance to pitting [33]. In particular a fine surface finish increases the corrosion resistance of the material and moves its pitting potential to more anodic potential values. The presence of both surface roughness and residual stress changes its electrochemical response [34, 35]. Furthermore, even though the roughness of the implant promotes better fixation, this can be detrimental to denture elements as it facilitates biofilm buildup that causes inflammation of the tissues [36].

Surface appearance of 310-H steel shows a striped surface which was made by the surface preparation process with some pits associated with these lines. Similar features were shown by stainless steels 304-H, 309-H, and 347-H. In the latter steel the presence of Nd-rich precipitates was observed but they did not affect its corrosion resistance. Small pits can be due to MnS particles in the alloy. It has been demonstrated that MnS inclusions perform as initiating sites for pitting [37]. The surface appearance shown by the 321-H stainless steel indicates the presence of additional corrosion processes to those observed in 310-H stainless steel. In this case, the presence of Ti-rich precipitates acting as cathode sites supports corrosion of the Fe-Cr-Ni matrix. In the area bounded by the dashed circle shown in Figure 13, the depression around the Ti-rich particles caused by the corrosion process created by the anode-cathode pair can be seen. In some areas this corrosion process caused the detachment of Ti-rich particles. Similar appearance was observed with the Mo-rich precipitates in 316-H stainless steel. The fact that the presence of the Nd-rich precipitates observed in the 347-H stainless steel did not increase its corrosion rate was possibly because the phases that form the anode-cathode pair have similar corrosion potential.

Austenitic stainless steels are essentially alloys of Fe-Cr-Ni, and the chromium addition has been known to improve their corrosion resistance. Nickel is the basic substitutional element used for austenite stabilization at all temperatures. Often, alloying elements, either interstitial such as C or N or substitutional such as Mo, Mn, Ti, and Nb, are used to obtain the required properties. However, the addition of alloying elements often results in the formation of carbides, nitrides, and intermetallic compounds. Molybdenum is a ferrite stabilizer and it also facilitates carbide precipitation. Stabilizing elements such as Nd and Ti greatly improve the creep strength of austenitic stainless steels, mainly by precipitating fine carbides intragranularly. These phases are not always desirable because they can provoke deterioration of mechanical or chemical properties. Nitrogen is a strong austenite stabilizer; it can act like carbon in stabilized stainless steels by precipitating in form of titanium or niobium nitrides [38]. It can be said that passivity of the austenitic stainless steels arises from the high corrosion resistance shown by the Cr(III) oxide-hydroxides present in the passivating layers. Chromium forms insoluble Cr_2_O_3_, which prevents the dissolution of iron. It has been reported that in solutions containing chloride ions Ni is rarely observed in the passive films of the stainless steels, and NiO is less effective than Cr_2_O_3_ in anchoring the water molecules and forming hydroxyl ions complex species [39].

However, according to the results shown here (Figures 12 and 13), it is evident that an increase in Ni content in stainless steels leads to an increase in corrosion rate. This may be due to the presence of a NiO layer on the passive (Cr_2_O_3_). This originates the presence of defects that favored the attack by chloride ions. It has been reported that, in NiO forming alloys, chloride ions can migrate through the passivating layer by replacing water and hydroxyl groups gathering in the metal oxide interface causing localized corrosion [10].

Figure 12 shows that in mouthwash solution the corrosion resistance of stainless steels increases with its chromium and nickel content but steels 316-H and 321-H do not obey this trend. Both steels have higher corrosion rates than expected according to Cr and Ni contents. Both steels contain additional elements such as Mo (316-H) and Ti (321-H). This suggests that the presence of these elements causes a detriment to corrosion resistance in artificial saliva environments. However, 347-H also contains an additional element (Nb) and its presence did not affect its corrosion resistance. As explained in the case of artificial saliva, this was possibly because the phases that form the anode-cathode pair have similar corrosion potentials. Analogous results were obtained in potentiodynamic polarization tests. However in this case the effect of Cr and Ni content was contrary to that observed in potentiodynamic tests. These analyses make clear that the corrosion behavior of materials is defined more consistently in long duration tests. Quick tests as potentiodynamic polarization curves are a tool that allows us to establish a relative corrosion resistance of materials but the initial trend may change in long time exposure because the corrosion process may cause changes in the local chemistry of the electrolyte making the environment more aggressive. Another consideration in long time tests is that changes occur when the residence time is long enough to reach a material-electrolyte thermodynamic equilibrium between the actual dynamic variables (*I*_corr_, *E*_corr_) and the corrosion process.

**Figure 14:**
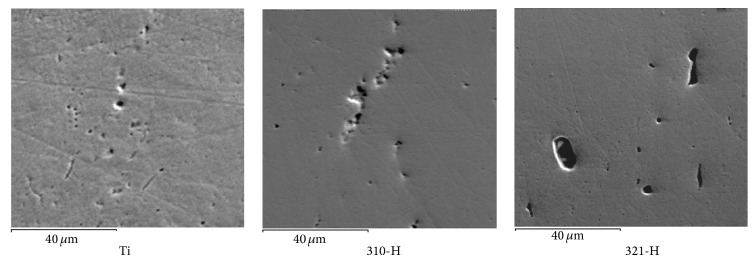
Superficial aspect of Ti, 310-H, and 321-H stainless steel after corrosion test in mouthwash solution.

Figure 14 shows the superficial aspect of titanium, 310-H, and 321-H stainless steel after the corrosion test in mouthwash solution. Surface appearance shown by Ti shows a homogeneous surface with isolated pits on its surface.

Surface appearance observed in 310-H steel shows a surface with isolated localized attack associated with surface lines originated by the specimen preparation process. Similar issues were shown by 304-H and 309-H steels. The surface appearance shown by stainless steel 321-H shows the presence of large voids corresponding to the spaces left by the detachment of Ti-rich precipitates. Similar features were observed for 306-H and 347-H steels. This indicates that the presence of precipitates containing Ti, Mo, and Nd acted as cathodic sites promoting corrosion of Fe-Cr-Ni matrix.

It is known that a biological environment can be highly aggressive for metallic materials, since some ions can cause localized corrosion much more favorably than others. The passive layers formed on the materials surface are usually assumed to be composed with an inner oxide layer, being the barrier layer inhibiting cations transfer, and an outer hydroxide being an exchange layer with the electrolyte. On the other hand, passivity of materials will depend on the ions present in the corrosive medium. In this sense it can be said that the aggressive ions may be categorized into two classes depending on the properties of their surrounding hydration shell. Chaotrope ions are ionic species referred to as water-structure breaking ions; they are usually large and generate weak electric fields around them, possessing a loose hydration shell which can be easily removed. The chaotrope ions are capable of initiating pitting (i.e., Cl^−^). On the other hand, the cosmotrope ions are ionic species referred to as water-structure-making ions and they exhibit a high surface charge density generating high electric fields at short distances such that they are able to bind water molecules in their vicinity more strongly than water itself. The cosmotrope ions do not break the passive layer and therefore do not initiate pitting (i.e., F^−^) [20]. This indicates that the surface features observed in the case of stainless steels in the presence of mouthwash are due primarily to the anode-cathode corrosion arising between the inclusions and Fe-Ni-Cr matrix.

In general, the passivity of the austenitic steels arises from the high corrosion resistance exhibited by the Cr(III) oxide-hydroxides present in the passivating layers. It is considered that chromium forms insoluble Cr_2_O_3_ which prevents the dissolution of iron. However, the addition of fluoride ions causes the surface films to become less stable. Fluoride ions are aggressive ions that degrade the protective oxide layer formed onto stainless steels surface. The complex formation of metal-fluoride molecules onto alloy surface can reduce the potential range for passivity and a further growth of the oxide film is hindered as the metal dissolution becomes the dominant electrochemical process [10]. In the presence of fluoride ion, the dissolution of the Cr_2_O_3_ may be due to the following reaction:

(2) $$\begin{array}{c} {\text{Cr}}_{2}{\text{O}}_{3}+{12\text{F}}^{-}+{8\text{H}}^{+}⟶{{2\text{CrHF}}_{6}}^{2-}+{3\text{H}}_{2}\text{O} \end{array}$$

The formation and repair of the anodic film could be according to the reaction:

(3) $$\begin{array}{c} 2\text{Cr}+{3\text{H}}_{2}\text{O}⟶{\text{Cr}}_{2}{\text{O}}_{3}+{6\text{H}}^{+}+{6\text{e}}^{-} \end{array}$$

However, the corrosion performance of stainless steels is also influenced by the presence of H_2_O_2_ in the mouthwash solution. Among many oxidants, hydrogen peroxide (H_2_O_2_) is quite unique. It has high active oxygen content (47%) and produces water as the only by-product [40, 41]. Reaction between H_2_O_2_ and Cr of the stainless steels is

(4) $$\begin{array}{c} 2\text{Cr}+{2\text{H}}_{2}{\text{O}}_{2}+{2\text{e}}^{-}⟶{\text{Cr}}_{2}{\text{O}}_{3}+{\text{H}}_{2}\text{O}+{2\text{H}}^{+} \end{array}$$

In relation to titanium, it has been reported that its corrosion in the presence of a solution with fluoride ions is superficial and transitional because the passive layer created on titanium surface reassembles quickly [42]. The negative influence of fluoride on the corrosion of titanium and its alloys has been reported earlier [7–9]. The surface of Ti consists of a thin oxide layer (2–6 nm), composed mainly of TiO_2_, and its corrosion is due to the alteration of the passivation oxide film [43]. TiO_2_-based passive film is destroyed by the attack of fluoride ions via the formation of a soluble Ti-F complex compound, [TiF_6_]^2−^ that form soluble salts [44–46]. Once the passive oxide layer on Ti is destroyed by fluoride attack, the rate of regeneration of passive oxide layer is a function of the dissolved oxygen concentration [17]. In the presence of fluoride ion, the dissolution of the Ti passive layer may be due to the following reaction:

(5) $$\begin{array}{c} {\text{TiO}}_{2}+6{\text{F}}^{-}+{4\text{H}}^{+}⟶{{\text{TiF}}_{6}}^{2-}+{2\text{H}}_{2}\text{O} \end{array}$$

The formation and repair of the anodic film of TiO_2_ could be according to the reaction:

(6) $$\begin{array}{c} \text{Ti}+2{\text{H}}_{2}\text{O}⟶{\text{TiO}}_{2}+{4\text{H}}^{+}+{4\text{e}}^{-} \end{array}$$

When Ti is brought into contact with H_2_O_2_ two phenomena will take place at the metal interface: (i) the Ti-catalyzed decomposition of hydrogen peroxide and (ii) the corrosion of the metal which will involve the metal dissolution into the electrolyte and the formation of a titanium oxide. This oxide is far thicker than the one obtained by simple immersion of the metal in saline solutions for similar periods and is composed of two layers: an outer layer highly porous and hydroxylated and an inner layer, much thinner and with insulating characteristics [18]. Reactions between H_2_O_2_ and titanium are [47]

(7) 2TiO2+H2O2+2e−Ti2O3+H2O+O2

(8) Ti2O3+H2O2⟶2TiO2+H2O+2e−

Therefore, the increase in *E*_corr_ and decrease in *I*_corr_ values observed in Figures 9 and 11 may be due to the presence of H_2_O_2_. Because H_2_O_2_ is a strong oxidizer, its presence in the mouthwash solution could have some effects on the properties of the passive film formed on Ti. The potential shift to the positive direction was observed in a similar study and it was attributed to an enhanced oxide film growth rate [47].

## 4. Conclusions

Potentiodynamic polarization tests in artificial saliva show that stainless steels have corrosion rates higher than titanium. However in mouthwash its behavior was reversed. In general it was observed that in the short-term tests corrosion resistance of stainless steels increases with increasing Cr and Ni and the presence of precipitates (Ti-rich, Nd, or Mo) increases its corrosion rate.

In artificial saliva stainless steels have a high susceptibility to pitting and its pitting potential increases by increasing the content of Cr, Ni, and Ti. In mouthwash the pitting potential of steels is only slightly affected by the contents of Cr and Ni and no significant influence was observed by the presence of Ti, Mo, and Nd.

The long-term tests in artificial saliva indicate that corrosion resistance of steel decreases by increasing its content of Cr and Ni and that the presence of precipitates rich in Ti and Mo increases the corrosion rate. In mouthwash the corrosion resistance of steels increases with increasing contents of Cr and Ni but precipitates of Ti, Mo, and Nd increase its corrosion rate. Precipitated phases function like cathodes favoring corrosion of Fe-Cr-Ni matrix which acts as the anode. The above takes place until the precipitates detach leaving large voids on the surface.

## Figures and Tables

**Table 1:** Chemical composition of austenitic stainless steels evaluated as reported by ASTM A213/A213M [11].

Grade	UNS designation	Chemical composition									
		C	Mn	P	S	Si	Cr	Ni	Mo	Nb	Ti
TP304H	S30409	0.04–0.10	2.00	0.045	0.030	1.00	18.0–20.0	8.0–11.0			
TP309H	S30909	0.04–0.10	2.00	0.045	0.030	1.00	22.0–24.0	12.0–15.0			
TP310H	S31009	0.04–0.10	2.00	0.045	0.030	1.00	24.0–26.0	19.0–22.0			
TP316H	S31609	0.04–0.10	2.00	0.045	0.030	1.00	16.0–18.0	11.0–14.0	2.00–3.00		
TP321H	S32109	0.04–0.10	2.00	0.045	0.030	1.00	17.0–19.0	9.0–12.0			4(C + N) − 0.7
TP347H	S34709	0.04–0.10	2.00	0.045	0.030	1.00	17.0–19.0	9.0–12.0		8C − 1.10	

**Table 2:** Chemical composition of artificial saliva (pH = 6.5) [12].

Compound	Content [g/L]
NaCl	0.600
KCl	0.720
CaCl_2_·2H_2_O	0.220
KH_2_PO_4_	0.680
Na_2_HPO_4_·12H_2_O	0.856
KSCN	0.060
NaHCO_3_	1.500
Citric acid	0.030

**Table 3:** Electrochemical parameters of materials in artificial saliva at 37°C.

Material	*E*_corr_ (mV)	*B*_*a*_ (mV/Dec)	*B*_*c*_ (mV/Dec)	*I*_corr_ (mA/cm^2^)
304-H	−252	2248	104	0.00090
309-H	−280	8572	138	0.0015
310-H	−268	965	111	0.00075
316-H	−292	1000	127	0.00176
321-H	−227	493	137	0.00148
347-H	−189	245	129	0.00083
Titanium	−159	260	286	0.00019

**Table 4:** Electrochemical parameters of the materials evaluated in mouthwash at 37°C.

Material	*E*_corr_ (mV)	*B*_*a*_ (mV/Dec)	*B*_*c*_ (mV/Dec)	*I*_corr_ (mA/cm^2^)
304-H	317	581	133	0.001713
309-H	330	356	129	0.00111
310-H	333	306	121	0.00127
316-H	302	642	140	0.0023
321-H	304	333	138	0.0027
347-H	310	371	126	0.0021
Titanium	−96	356	176	0.0116
